# Faecal inflammatory protein markers in children with autism spectrum disorder are comparable to their healthy siblings

**DOI:** 10.3389/fpsyt.2026.1792801

**Published:** 2026-04-15

**Authors:** Joško Osredkar, Petra Finderle, Uroš Godnov, Maja Jekovec-Vrhovšek, Veronika Vidova, James Price Elliott, Teja Fabjan, Gorazd Avguštin, Damjan Osredkar, Kristina Kumer

**Affiliations:** 1 Institute of Clinical Chemistry and Biochemistry, University Medical Centre Ljubljana, Ljubljana, Slovenia; 2 Faculty of Pharmacy, University of Ljubljana, Ljubljana, Slovenia; 3 The Faculty of Mathematics, Natural Sciences and Information Technologies, University of Ljubljana, Koper, Slovenia; 4 Center for Autism, Unit of Child Psychiatry, University Children’s Hospital, University Medical Centre Ljubljana, Ljubljana, Slovenia; 5 Research Centre for Toxic Compounds in the Environment (RECETOX), Faculty of Science, Masaryk University, Brno, Czechia; 6 Environmental Exposure Assessment Research Infrastructure-Czech Republic (EIRENE-CZ), Brno, Czechia; 7 Department of Microbiology, Biotechnical Faculty, University of Ljubljana, Domžale, Slovenia; 8 Division of Pediatrics, Department of Child, Adolescent and Developmental Neurology, University Medical Centre Ljubljana, Ljubljana, Slovenia; 9 Faculty of Medicine, University of Ljubljana, Ljubljana, Slovenia

**Keywords:** alpha-1-antitrypsin, autism spectrum disorder, calprotectin, immunoglobulin A, siblings, stool samples

## Abstract

**Background:**

Autism spectrum disorder (ASD) is a complex neurodevelopmental condition often accompanied by gastrointestinal (GI) symptoms. Inflammatory proteins in stool have been proposed as potential biomarkers, but evidence remains inconsistent. We compared fecal levels of α1-antitrypsin (A1AT), immunoglobulin A (IgA), and calprotectin (Cal) in 57 children with ASD and 57 biological siblings without ASD. Sibling designs are now preferred to disentangle ASD-specific biology from shared environmental and microbiome factors. Participants were carefully screened to exclude recent antibiotic use, digestive problems, gastrointestinal infections, and abnormal dietary patterns, thereby controlling for major factors known to influence gut inflammatory markers.

**Methods:**

Stool samples were thawed, freeze-dried, and proteins extracted using ammonium bicarbonate buffer with sodium deoxycholate. After BCA quantification, samples were reduced, alkylated, spiked with stable isotope–labelled peptides, and digested with trypsin. Peptides were purified and analyzed by UHPLC–MS/MS (Agilent 6495A) in dynamic SRM mode. Quantification used internal standards and normalization to total protein. Ratios of IgA1/IgA2 and S100A8/S100A9 were calculated. ASD severity was evaluated using the Childhood Autism Rating Scale (CARS).

**Results:**

Children with ASD showed trends toward higher IgA and calprotectin and lower α1-antitrypsin compared with siblings, but differences were not statistically significant. Subgroup analysis suggested different distribution patterns in moderate versus severe ASD, including higher IgA in the moderate group and altered S100A8/S100A9 ratio in the severe group. These subgroup findings were exploratory, derived from critically underpowered *post-hoc* analyses (severe subgroup: n = 11 pairs, ~18% power for medium effects), and should be considered hypothesis-generating only, pending validation in adequately powered pre-registered studies.

**Conclusions:**

The results are consistent with recent meta-analyses reporting no consistent evidence of gut inflammation in ASD. Larger, sex-matched studies with full assay validation are needed to clarify the role of stool proteins in ASD.

## Introduction

Autism Spectrum Disorder (ASD) is a neurodevelopmental disorder that affects the way individuals interact and communicate. ASD impacts at least 1 out of every 59 children in the U.S. (1), although this is likely underestimated (2). The exact origin of ASD is unknown, with a high hereditary prevalence and diverse developmental trajectories evidencing environmental influence (3–5). ASD is typically diagnosed during childhood and is characterized by differences in the brain. Severity is based upon deficits in social communication, interaction and restricted repetitive patterns of behavior or interests (6). Biological markers to predict ASD development risk or forecast outcome trajectories could have great clinical utility: to assist in early diagnosis, aid prognosis and support the identification of potential therapeutic targets (7, 8). Gastrointestinal (GI) disorders are among the most common medical conditions comorbid with ASD (3, 9). Gut inflammatory processes have been implicated in the development of ASD, emphasizing the potential role of inflammation in the pathogenesis of ASD (10, 11). Furthermore, mucosal immune system differences are observed in some individuals with ASD, yet connection with GI disorders and role in the development of autism is not yet fully understood (12, 13). Immune/inflammatory marker proteins have been investigated as potential biomarkers for ASD (14). However, matched (age/gender) case-control or twin studies mostly focused on heritability (5), rarely incorporating measurement of protein and/or other potential molecular biomarkers (15). Via integrating multiple cohorts and multi-omic datasets, a functional architecture along the gut-brain axis was constructed that correlated to heterogeneity of ASD phenotypes when comparing children with ASD to unrelated age- and sex-matched neurotypical controls. Yet, the ASD-microbiome associated functional architecture was not observed in sibling-matched cohorts. Temporal associations of microbiome composition and ASD phenotype were observed, and the immune system was identified as pivotal in mediating the communication between the gut microbiome and the human brain. In particular, pro-inflammatory cytokines correlated to microbial taxa elevated in children with ASD (16) and their differentials potentially associate to specific symptoms (17).

On the other hand, a metanalysis of protein fecal biomarkers did not find evidence to support higher GI inflammation in autistic children and adolescents compared to non-autistic controls. Yet, the authors note that the heterogeneity among studies leaves the possibility that there is a subset of children and adolescents with autism who exhibit higher GI inflammation (18).

Alpha-1-antitrypsin (A1AT), immunoglobulin A (IgA) and calprotectin (Cal) are the primary antibodies released by intestinal immune cells and have been assayed in stool to study perturbed intestinal homeostasis (19), indicative of gut protein leakage, mucosal immunity, and neutrophil-driven inflammation, respectively.

A1AT of the serine protease inhibitor (SERPIN) family plays an important role in regulating protease activity in various tissues, including the lungs, liver, and gastrointestinal tract. Specifically, A1AT has a crucial role in protecting the body from the damaging effects of neutrophil elastase, an enzyme released by white blood cells to fight infection and critical to immune function (20). Fecal concentrations of A1AT isoform 1 (A1AT-1) are commonly used to assess protein leakage into the intestinal tract, particularly in the context of protein-losing enteropathies and gastrointestinal diseases (21). Associations between A1AT deficiency and autism have previously been observed though the relationship to GI symptoms not investigated (22).

IgA is essential for mucosal immunity in the gastrointestinal and respiratory tracts, acting as the first defense against pathogens and maintaining gut microbiota balance. IgA has two subclasses, IgA1 and IgA2, encoded by the IGHA1 and IGHA2 genes. These subclasses differ in structure, glycosylation patterns, and susceptibility to proteolytic cleavage, with IgA2 having stronger pro-inflammatory functions (23). Imbalances in IgA1 and IgA2 levels can affect mucosal immunity and homeostasis, highlighting their importance in the immune defense at mucosal surfaces (24).

Some studies report ASD children have significantly higher level of fecal IGHA1 than healthy children, potentially reflecting alterations in gut permeability and immune function (2). For example, Rose et al. (12) analyzed the stool samples of 53 ASD children and 34 healthy children and found that the levels of IGHA1 were significantly higher in the ASD group than in the healthy group, while the levels of IGHA2 were significantly lower in the ASD group. The authors suggest that the altered levels of these IgA subclasses may reflect differences in gut microbiota and immune function in ASD individuals. Contrastingly, other studies have observed no differences in fecal IgA (25). Even when differences are observed, it has been suggested that IgA is indicative of altered microbiome composition but does not distinguish between autistic children and adolescent with and without GI symptoms (26). Nevertheless, none of these investigations determined or assessed the IgA1:IgA2 ratio.

Cal is a heterodimeric complex formed by the association of two molecules of S100A8 with one molecule of S100A9. Cal is released upon the activation of neutrophilic granulocytes. The incidence of Cal in feces indicates neutrophil infiltration into the intestinal tract, and the level of Cal is associated with macroscopic and histological inflammation. Cal is stable in feces and can be measured as a marker of intestinal inflammation-induced leaky gut (18). The biological roles of the Cal subunits S100A8 and S100A9 during the coordination of an inflammatory response at mucosal surfaces throughout organ systems have been elucidated by recent investigations (27). The ratio of S100A8 and S100A9 components can vary in different physiological and pathological conditions (28). Measuring the individual concentrations of S100A8 and S100A9, as well as their ratio, can provide valuable insights into the underlying inflammatory processes and aid in the diagnosis, monitoring, and understanding of various diseases. Whilst a meta-analysis of fecal Cal showed no evidence of alterations between autistic and non-autistic children and adolescent (18), the individual levels of S100A8 and S100A9 in neurologic diseases, including autism spectrum disorder (ASD), is relatively limited compared to their studies in other inflammatory conditions.

Recent multi-level analyses of the microbiome–gut–brain axis in ASD have shown that associations observed when comparing ASD children to unrelated neurotypical controls are markedly attenuated when sibling-matched cohorts are used, suggesting that shared familial, environmental, and microbiome factors account for a substantial portion of the signal (16, 29, 30, 31). Sibling designs are therefore increasingly recognized as a robust strategy to disentangle ASD-specific biology from shared household and genetic influences, particularly for gut-related biomarkers.

Given the conflicting evidence from prior studies and recent meta-analyses reporting largely null findings (18), we hypothesized that when genetic and environmental confounders are controlled through sibling matching, stool inflammatory protein differences between ASD and non-ASD children would be minimal or absent. To test this hypothesis, we designed a sibling-controlled study with three specific aims:

Primary Aim: To compare fecal concentrations of α1-antitrypsin, immunoglobulin A (total, IgA1, IgA2), and calprotectin (S100A8, S100A9, total) between children with ASD and their biological siblings, controlling for shared genetic and environmental factors.

Secondary Aim: To examine whether protein subclass ratios (IgA1/IgA2 and S100A8/S100A9) differ between groups, as ratios may reveal functional immune perturbations not apparent in absolute concentrations.

Exploratory Aim: To investigate whether inflammatory protein patterns differ according to ASD symptom severity, as assessed by the Childhood Autism Rating Scale (CARS), to explore potential severity-based subgroups.

We employed rigorous screening to exclude confounding factors (recent antibiotic use, gastrointestinal symptoms, dietary abnormalities) and state-of-the-art UHPLC-MS/MS quantification with stable isotope-labeled internal standards. By using a sibling-based design with stringent inclusion criteria and advanced analytical methods, this study seeks to clarify whether stool inflammatory proteins represent valid biomarkers for ASD or whether previously reported associations reflect uncontrolled confounding. In addition to α1-antitrypsin, IgA, and calprotectin, we also assessed three eosinophil-derived inflammatory proteins: eosinophil-derived neurotoxin (EDN), myeloperoxidase (MPO), and eosinophilic cationic protein (ECP). These markers reflect eosinophil activation and degranulation and have been examined in allergic and inflammatory gastrointestinal conditions. While literature on these markers in ASD is limited, eosinophilic infiltration has been reported in some gastrointestinal biopsies from ASD children (32, 33). We therefore included these markers in our comprehensive panel to provide exploratory data on eosinophil-mediated inflammation in ASD.

A description of the individual parameters, their physiological role and their association with different diseases and ASD is shown in **Table 1**.

**Table 1:** Physiological role of inflammatory proteins and association with various diseases and ASD.

Parameter	Physiological role	Pathologies	In ASD
A1AT	Alpha-1 antitrypsin is a 52 kDa serine protease inhibitor that is abundant in serum. Its primary function is to inhibit neutrophil elastase, preventing the breakdown of elastin in the lung tissue.	A1AT deficiency can lead to emphysema and liver disease	Potential abnormal levels indicating inflammation or immune dysregulation
IgA	Immunoglobulin A (IgA) is an antibody that plays a crucial role in the immune function of mucous membranes. It exists in two forms, Serum IgA and Secretory IgA. The latter is the main immunoglobulin found in mucous secretions.	High levels can indicate an autoimmune or inflammatory condition	Potential abnormal levels indicating inflammation or immune dysregulation
Cal	Calprotectin is a 36.5 kDa protein complex found in neutrophil cytosol, composed of two proteins, S100A8 and S100A9, in a (S100A8/S100A9)2 tetramer. It has antimicrobial activity against bacteria and fungi.	High levels can indicate inflammatory bowel disease (IBD)	Potential abnormal levels indicating inflammation or immune dysregulation

## Materials and methods

### Patients

Our study population consisted of 57 children with ASD (48 boys/9 girls) and 57 healthy children (29 boys/28 girls) who were their biological siblings, recruited at the Centre for Autism, University Children’s Hospital, Ljubljana, between 2016 and 2020. The study protocol was approved by the National Medical Ethics Committee (0120-201/2016–2 KME 78/03/16).

### Inclusion criteria

For ASD group:

1. 1Age 0.9-17.0 years at time of sample collection
2. Diagnosed with ASD by multidisciplinary team (paediatrician, neuropsychiatrist, psychologist) according to DSM-5 criteria
3. At least one biological sibling willing to participate

For sibling control group:

1. Biological sibling of an ASD participant
2. No ASD diagnosis
3. No chronic gastrointestinal or systemic disease
4. Not on long-term medication (as confirmed by parental report)

### Exclusion criteria (applied to both groups)

Before enrolment, parents/guardians of both ASD children and their siblings were systematically interviewed regarding the following exclusion criteria:

1. Antibiotic exposure: Receipt of any antibiotic medication within 6 months prior to stool sample collection (to avoid confounding from antibiotic-induced microbiota disruption)
2. Gastrointestinal symptoms: Presence of any active digestive problems including but not limited to: chronic diarrhea, constipation requiring medical intervention, abdominal pain, blood in stool, or diagnosed inflammatory bowel disease
3. Acute gastrointestinal infections: Any diagnosed gastrointestinal infection (viral, bacterial, or parasitic) within 3 months prior to sample collection
4. Dietary abnormalities: Parental report of highly restrictive diets (e.g., elimination diets excluding multiple food groups), feeding tubes, or other abnormal dietary patterns that could confound inflammatory marker interpretation
5. Chronic medication use (siblings only): Regular use of medications known to affect GI inflammation (e.g., NSAIDs, corticosteroids, proton pump inhibitors)

Children meeting any exclusion criterion were not enrolled. This screening process was designed to minimize the influence of known factors that could affect stool inflammatory protein levels independent of ASD diagnosis. These “known factors” refer to well-established confounders of fecal inflammatory markers documented in gastroenterological literature:

- Antibiotics: Alter gut microbiota composition and immune responses for up to 6 months post-treatment
- Active GI inflammation: Elevates IgA, calprotectin, and α1-antitrypsin independently of ASD status
- Dietary composition: High-fat/low-fiber diets alter microbiota and inflammatory tone; restrictive elimination diets common in ASD could confound findings
- Medications: NSAIDs increase intestinal permeability and inflammatory markers

By systematically excluding children with these exposures from both ASD and control groups, we aimed to isolate ASD-associated differences from these well-characterized confounding influences while preserving the advantages of sibling matching for genetic and familial environmental factors.

Healthy siblings were matched to cases by age, with an average difference of 1.3 years (SD 3.5). Due to the familial design, sex matching was not feasible and is acknowledged as a study limitation. The demographic features of the ASD and control groups are given in **Table 2**.

**Table 2:** Description of patients and controls used in this study.

Item	Control (N = 57)	ASD (N = 57)	Difference	p-value	Test
Sex					
Boys	29 (50.9%)	48 (84.2%)	3 (10.3%)	<0.001	Chi-square
Girls	28 (49.1%)	9 (15.8%)	22 (78.6%)		
Age					
Mean (SD)	9.02 (3.62)	10.36 (3.35)	1.34 (3.49)	0.01	Paired t-test
Median (Q1, Q3)	8.70 (6.40, 11.20)	10.10 (7.90, 13.00)	2.10 (-1.40, 3.70)		
Min-Max	0.90–16.70	4.20–17.00	-10.00–7.80		

A1AT, α1-antitrypsin; ASD, autism spectrum disorder; Cal, calprotectin; CARS, Childhood Autism Rating Scale; IgA, immunoglobulin A; Q1, first quartile; Q3, third quartile; SD, standard deviation.

ASD severity was assessed using the Childhood Autism Rating Scale (CARS), a 15-item behavioral rating scale designed to identify children with autism and determine symptom severity. Each item is scored on a scale from 1 (normal for age) to 4 (severely abnormal), yielding total scores ranging from 15 to 60. CARS scores are interpreted as follows: 15-29.5 indicates no autism, 30-36.5 indicates mild to moderate autism, and ≥37 indicates severe autism (34). For the present study, we used a cutoff of 36 to stratify participants into two subgroups: CARS <36 (mild/moderate symptoms) versus CARS >36.5 (severe symptoms). This dichotomization was chosen *post-hoc* to explore whether inflammatory protein patterns differ by severity, based on the clinically established CARS threshold distinguishing mild-moderate from severe presentations. We acknowledge this categorization was not pre-specified and represents exploratory analysis.

The ASD group was significantly older than the sibling control group (mean 10.36 vs. 9.02 years; paired t-test p = 0.01), with a mean within-pair age difference of 1.34 years (SD 3.49). This age difference reflects that ASD diagnosis typically occurs at ages 3-5, and sibling recruitment occurred concurrently, resulting in some siblings being younger. The sex distribution differed significantly between groups (χ² = 21.3, p <0.001), with 84.2% males in the ASD group versus 50.9% in the sibling group, reflecting the well-established male predominance in ASD diagnoses (approximately 4:1 male-to-female ratio). As participants were biological sibling pairs, they shared household environment, parental socioeconomic status, and genetic background by study design, minimizing potential confounding from these factors.

### Stool sample collection

Parents/guardians are given detailed instructions on how to take samples when they sign the informed consent. Information on how to collect, handle, and store stool samples is provided in the **Supplementary Protocol** for stool sampling.

Once the stool samples were collected in the packaging previously provided to the laboratory, the sample was brought to the laboratory as soon as possible. If they were unable to do so on the same day due to the distance to the laboratory, the samples were frozen at -20°C. The maximum length of time at -20 was 48 hours When the samples were received in the laboratory, they were frozen at minus 80°C. Transport to the laboratory where the analyses were carried out was on dry ice.

### Sample collection quality control and variability assessment

To assess the impact of home-based collection variability, we examined several quality metrics. Inter-individual coefficients of variation (CV) in the control group were: IgA total 77.7%, IgA1 101.4%, IgA2 77.5%, α1-antitrypsin 45.6%, S100A8 69.5%, S100A9 67.6%, and calprotectin 64.8%. These high CVs reflect true biological variability combined with collection/storage variability. In contrast, intra-assay analytical variability was well-controlled (mean CV 12.9% across triplicate measurements), indicating that technical measurement error contributed minimally to overall variability.

Sensitivity analyses were conducted excluding extreme outliers (>3 standard deviations from mean). Primary findings (null results for main comparisons) did not substantively change when outliers were excluded (all p-values remained >0.05), suggesting results are robust to extreme values. However, we cannot quantify the specific contribution of home-collection timing variability to overall variance. Stool samples were collected by families at variable times before freezing (up to 48 hours at -20°C per protocol). While we provided detailed written instructions to minimize variability, we cannot exclude that variable compliance with collection protocols contributed to measurement variability and reduced our power to detect true group differences.

### Stool sample preparation

Protein analysis was performed via a previously validated assay to quantify immunomarkers in neonatal fecal swabs (19), with modification for adult stools swabs inclusion of winged peptides (35) as described in Biomarker Analytical laboratories (36). SOP for quantification of immunity marker proteins in fecal swabs using LC-MS/MS (1.0.0). Zenodo, https://zenodo.org/records/7625076,” 2025). Stool samples were removed from -80°C freezer and thawed on ice for 1 hour. Thawed stool samples were collected using a pre-weighed broken FLOQ swab (Copan Diagnostics, USA, cat# 520CS01) into a homogenization vial (Benchmark Scientific, USA, cat# D1031-T20) and weight of sample measured. Samples were closed with aluminum foil and placed into -80°C for 30 min prior to freeze drying overnight. Dried stool samples were weighed, sealed with screw lid, and kept at -80°C until extraction. The wet weight, dry weight, and water content of stool samples were calculated.

Proteins were extracted from dried stool sample using protein extraction buffer content of 50 mM Ammonium bicarbonate with 5g/L sodium deoxycholate. A 1 mL of protein extraction buffer was added to vial and proteins were extracted using a homogenizer with setting: 4 pulses × 10 s; 4 m/s; inter-time 10 s; lab temperature. Extracts were centrifuged at 13–000 RPM for 5 min at 8°C and 500 µL supernatant transferred to a new 96-deep well plate (Costar, cat. #3959) and stored at -80°C until further analysis.

The 96-well plate with protein extract was taken out of the freezer and thawed at RT for 90 min. Total protein concentration was measured using BCA kit (Thermo Scientifics, cat# 23225). Samples were diluted 10-fold before BCA analysis via taking 10 µL into a microplate and adding 90 µL of protein extraction buffer. Samples were diluted to achieve total protein concentration up to 250 µg/sample. Thus a 50, 30 or 20 µL of protein extract was taken for following sample preparation. Extraction buffer was added to samples with 20 and 30 µL to achieve a volume 50 µL for all samples. Proteins in the samples were then reduced using 5 µL of 200 mM dithiothreitol for 10 min at 90°C. After cooling, the proteins were alkylated using 5 µL of 400 mM iodoacetamide and incubated for 30 min in dark. A 10 µL of 500 nM stable isotope labeled (SIL) peptides mixture was then added to sample. A list of SIL peptides is shown in **Supplementary Table 1**. Reduced and alkylated proteins were digested adding 3 µL of 1 µg/µL trypsin solution. Samples were properly sealed and placed into shaking incubator heated at 37°C for 5 hours (Biosan, Latvia, ES-20). The digestion was stopped adding 200 µL of 2% formic acid (FA). Digested samples were processed at SPE Oasis prime HLB 96 well plate, 30 mg (Waters, USA, cat# 186008054). Samples were directly loaded on SPE and washed with 300 µL of 2% FA. Peptides were eluted into a new 96 well plate with V bottom (Waters, cat#186005837) using 50% acetonitrile (ACN) with 2% FA. Then the samples were dried out in vacuum evaporator and dissolved in 5% ACN with 0.1% FA.

### Mass spectrometry analysis

Samples were analyzed on a UHPLC system (1260 series Agilent, CA) coupled with a triple quadrupole mass spectrometer (AJS 6495A, Agilent, CA). Samples were injected (2 µL) on the analytical column (C18 Peptide CSH; 1.7 µm, 2.1 mm i.d. × 100 mm; cat. #186006937; Waters, MA). The column temperature was held at 40°C. The mobile phase consisted of solution A (0.1% FA in water) and solution B (0.1% FA in 95% ACN). The flow rate was 300 µL/min. The gradient elution program consisted of analytical (0–30.9 min) and re-equilibration part (31–35 min): 0.0 min 5% B; 25 min 30% B; 25.5 min 95% B; 30.9 min 95% B; 31 min 5% B; 35 min 5% B. A standard-flow electrospray source operated in positive ion mode (capillary voltage 3.5 kV; gas flow rate 18 L/min at 200°C; sheath gas flow 12 L/min at 350°C; nozzle voltage 500 V). We monitored 78 transitions in the dynamic SRM mode analysis, with 3 min window scheduled around peptide experimental RT. SRM signature transitions were equivalent for proteotypic peptide and corresponding SIL internal standard, i.e., a single SRM quantifier transition and 2 additional qualifier SRM transitions were acquired and are listed in **Supplementary Table 2**.

### Quality control

The UHPLC-MS sequence included analysis of instrumental blanks (after every 3 samples) and solvent mixture of peptides analyzed at start and end of acquisition, plus approximately after every 30 samples. Samples were measured in triplicate and the total average coefficient of variation (CV) across replicates was 12.9% for all peptides. Measures are reported as nanomolar peptide concentrations, and recalculated to protein weight and then normalized to total protein content.

### Statistics

R version 4.3.1 in conjunction with RStudio version 2023.12.0 was used for statistical analysis., using the tidy verse suite (37) for visualization and arsenal package (38) to compare groups. The Shapiro-Wilk tests from stats package (39) was used to evaluate the distribution of data. Results showed that all datasets had non-normal distributions and so the non-parametric Wilcoxon signed-rank test was employed for comparisons. The Benjamini-Hochberg procedure was used to control false discovery rate, with alpha significance level of 0.05. IgA1/IgA2 and S100A8/S100A9 ratios were calculated for both groups of children (according to diagnosis and CARS score).

#### Outlier detection and handling

We analyzed paired data and maintained the pair structure throughout all analyses. To ensure robust statistical inference unaffected by extreme values, we applied Tukey’s interquartile range (IQR) rule to detect outliers separately for each biomarker parameter. For each protein marker, values falling outside the range [Q1 – 1.5×IQR, Q3 + 1.5×IQR] were flagged as potential outliers using the full sample (N = 114 individuals) for that specific parameter.

Critically, when either member of a sibling pair was flagged as an outlier on a given parameter, we removed the entire pair from analysis of that parameter only to preserve the paired structure of the data. This pair-wise removal was applied independently for each biomarker. Consequently, different parameters have different numbers of pairs available for analysis:

• IgA1: 52 pairs (5 pairs removed due to outliers)
• IgA2: 49 pairs (8 pairs removed)
• IgA total: 51 pairs (6 pairs removed)
• α1-antitrypsin: 53 pairs (4 pairs removed)
• S100A8: 44 pairs (13 pairs removed)
• S100A9: 44 pairs (13 pairs removed)
• Calprotectin: 44 pairs (13 pairs removed)

Because outlier detection was applied separately to each marker, a given pair might be excluded from analysis of one protein but retained for another. This parameter-specific approach maximizes statistical power by retaining all valid pairs for each comparison.

#### Rationale for statistical test selection

We considered linear mixed-effects models (LMMs) as an alternative analytical approach, given their ability to handle unbalanced data and retain unpaired observations when a sibling’s data is removed as an outlier. However, we chose the Wilcoxon signed-rank test for the following reasons: (1) all biomarker distributions were significantly non-normal (Shapiro-Wilk p < 0.05), and while LMMs can accommodate non-normality through transformations, the Wilcoxon test requires no distributional assumptions; (2) our study design is fundamentally paired, and the Wilcoxon test directly evaluates within-pair differences; (3) LMMs require assumptions about the distribution of random effects that are difficult to verify with our sample size. As a sensitivity analysis, we repeated all primary comparisons including all 57 pairs without outlier removal; results were consistent with the primary analysis (all p-values > 0.05), indicating that our null findings are robust to the outlier handling approach. We acknowledge that the pair-wise outlier removal strategy reduced effective sample sizes (most notably for S100A8, S100A9, and calprotectin, where 13 pairs were excluded), and future studies with larger sample sizes should consider LMM-based approaches to maximize statistical power (40).

#### Power calculations

*Post-hoc* power calculations were conducted using G*Power 3.1.9.7 for Wilcoxon signed-rank tests (two-tailed, α=0.05). With 57 paired observations, the study had approximately:

• 70% power to detect medium effect sizes (Cohen’s d ≈ 0.5)
• 95% power to detect large effects (d ≈ 0.8)
• <30% power to detect small effects (d ≈ 0.2)

For severity-stratified subgroups, power was substantially reduced:

• Mild/moderate subgroup (n=27 pairs): ~35% power for medium effects
• Severe subgroup (n=11 pairs): ~18% power for medium effects

These power limitations substantially restrict interpretation of subgroup findings.

#### Multiple comparisons correction

For exploratory severity-stratified analyses, we applied Benjamini-Hochberg false discovery rate (FDR) correction to control for multiple comparisons. Both unadjusted and FDR-adjusted p-values are reported.

### Data availability statement

The mass spectrometry datasets generated and analyzed during the current study are publicly available in the MassIVE repository at https://doi.org/10.6069/y9hr-js96. Statistical analysis code (R scripts using R version 4.3.1 and RStudio version 2023.12.0) is available from the corresponding author upon reasonable request. Sex-stratified analyses were conducted but are not presented in supplementary materials due to insufficient statistical power (particularly for females, n=9 ASD vs 28 siblings). These exploratory sex-stratified results are available from the corresponding author upon reasonable request. This study was conducted in accordance with the Declaration of Helsinki and was approved by the National Medical Ethics Committee (0120-201/2016–2 KME 78/03/16; 3 February 2021) and by the Slovenian Agency for Research and Innovation (Protocol J3-1756, June 2019). Written informed consent was obtained from all parents/guardians of participating children prior to enrolment. All procedures were conducted in accordance with the Declaration of Helsinki and relevant national regulations.

## Results

The number of sibling pairs available for each parameter comparison varied (N = 36 to N = 54) due to parameter-specific outlier removal as described in Methods. This variability does not reflect missing data or selective exclusion, but rather a systematic, pre-specified statistical procedure to ensure robust inference by removing extreme values while maintaining paired data structure.

Eosinophil-derived neurotoxin (EDN), myeloperoxidase (MPO), and eosinophilic cationic protein (ECP) were included in our analytical panel to assess eosinophil-mediated inflammation. However, these markers were below the limit of detection (LOD) in the majority of samples. The LOD and LOQ values, determined from signal-to-noise ratios in our UHPLC-MS/MS SRM analysis (LOD = 3× S/N; LOQ = 10× S/N), were as follows: EDN (LOD = 0.05 nM, LOQ = 0.15 nM), MPO (LOD = 0.08 nM, LOQ = 0.25 nM), and ECP (LOD = 0.04 nM, LOQ = 0.12 nM). EDN was undetectable (<LOD) in 89% (102/114) of samples, MPO in 84% (96/114), and ECP in 91% (104/114).In the few individuals with detectable levels (n=8–12 per marker), concentrations were below the limit of quantification (LOQ), precluding reliable quantitative analysis. The very low prevalence of detectable eosinophil markers suggests minimal eosinophilic gastrointestinal inflammation in both ASD children and their siblings in our cohort, which had been carefully screened to exclude active GI symptoms. Consequently, these three proteins were excluded from statistical analysis, and subsequent results focus on the neutrophil/lymphocyte markers (IgA, α1-antitrypsin, calprotectin) that were reliably quantifiable.

### Individual inflammatory protein markers

**Figure 1:**
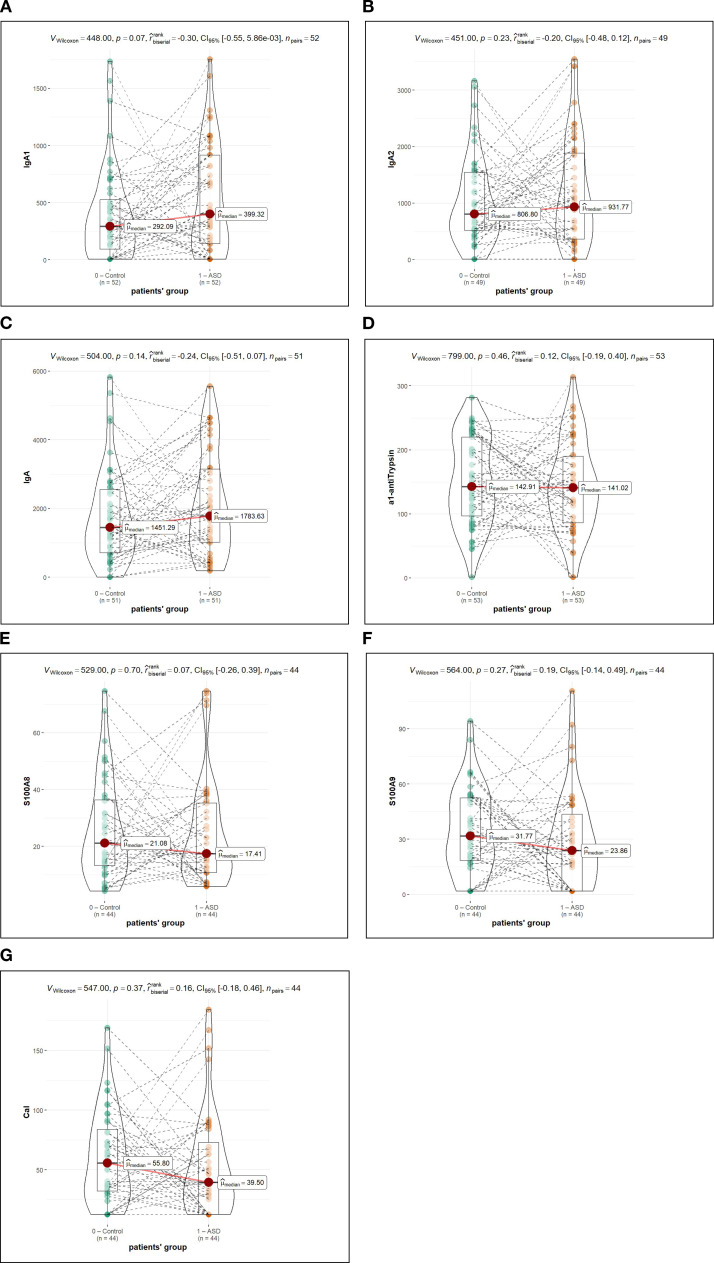
Violin plots showing the median values and statistical significance between the two groups. 0, control group; 1, children with ASD. **(A)** refers to IgA1; **(B)** to IgA2; **(C)** to IgA; **(D)** to A1AT-1; **(E)** to S100A8; **(F)** to S100A9 and **(G)** to Cal. All values are expressed as ng/mg of protein.

Faecal levels of A1AT-1, IgA (total), IgA1, IgA2, S100A8, S100A9, and Cal showed no statistically significant differences between children with ASD and their siblings (**Table 3**). Children with ASD had numerically higher median IgA levels compared to controls (1783.63 ng/mg vs. 1451.29 ng/mg; p = 0.14). This pattern was consistent for both IgA subclasses: IgA1 (399.32 vs. 292.09 ng/mg; p = 0.07) and IgA2 (931.77 vs. 806.80 ng/mg; p = 0.23). A1AT-1 levels showed no meaningful differences (142.91 vs. 141.02 ng/mg; p = 0.46). S100A8 and S100A9, the component proteins of calprotectin, also did not differ significantly between groups (S100A8: 21.08 vs. 17.41 ng/mg, p = 0.70; S100A9: 31.77 vs. 23.86 ng/mg, p = 0.27), nor did total calprotectin (55.80 vs. 39.50 ng/mg; p = 0.37). Effect sizes were small across all comparisons (|r| < 0.30) (**Figure 1**, Table statistics).

**Table 3:** Levels of inflammatory proteins in stool samples of ASD children and their siblings.

ng/mg of protein	Control group	ASD group	N	p value
IgA1			52	0.07
Mean (SD)	395.26 (400.79)	532.99 (466.14)		
Median (Q1, Q3)	292.09 (91.57, 524.71)	399.32 (140.28, 914.89)		
Min - Max	5.30 - 1735.41	5.30 - 1756.08		
IgA2			49	0.23
Mean (SD)	1005.66 (779.24)	1209.20 (947.76)		
Median (Q1, Q3)	806.80 (518.11, 1542.89)	931.77 (361.76, 1880.85)		
Min - Max	6.39 - 3164.64	6.39 - 3549.96		
IgA			51	0.14
Mean (SD)	1745.48 (1355.98)	2107.34 (1499.28)		
Median (Q1, Q3)	1451.29 (705.94, 2556.75)	1783.63 (1020.18, 3145.70)		
Min - Max	3.36 - 5826.63	189.36 - 5559.23		
A1AT-1			53	0.46
Mean (SD)	149.76 (68.25)	143.06 (70.21)		
Median (Q1, Q3)	142.91 (96.99, 219.86)	141.02 (86.22, 190.05)		
Min - Max	1.33 - 281.33	1.33 - 313.78		
S100A8			44	0.70
Mean (SD)	25.42 (17.66)	24.25 (18.88)		
Median (Q1, Q3)	21.08 (13.16, 36.29)	17.41 (10.78, 35.23)		
Min - Max	4.22 - 74.67	5.83 - 74.69		
S100A9			44	0.27
Mean (SD)	34.26 (23.15)	28.62 (25.88)		
Median (Q1, Q3)	31.77 (18.39, 52.47)	23.86 (1.82, 43.51)		
Min - Max	1.82 - 94.25	1.82 - 110.54		
Cal			44	0.37
Mean (SD)	60.45 (39.18)	53.15 (43.56)		
Median (Q1, Q3)	55.80 (32.18, 83.79)	39.50 (12.61, 72.86)		
Min - Max	12.61 - 168.92	12.61 - 184.23		

### Protein ratio analysis

The calculated IgA1/IgA2 ratio showed no significant difference between ASD and control groups (0.36 vs. 0.33; p = 0.93). The S100A8/S100A9 ratio similarly did not differ significantly in the overall analysis (0.74 vs. 0.76; p = 0.15). These ratios are presented in **Table 4** with associated statistical values.

**Table 4:** Protein concentration ratios of Cal and IgA components in ASD children compared to their siblings.

Protein ratio	Control group	ASD group	N	p value
S100A8/S100A9			36	0.15
Mean (SD)	0.71 (0.14)	0.75 (0.13)		
Median (Q1, Q3)	0.74 (0.60, 0.80)	0.76 (0.66, 0.82)		
Min - Max	0.38 - 0.93	0.44 - 0.97		
IgA1/IgA2			54	0.93
Mean (SD)	0.40 (0.28)	0.40 (0.28)		
Median (Q1, Q3)	0.36 (0.18, 0.60)	0.33 (0.19, 0.56)		
Min - Max	0.00 - 1.10	0.01 - 1.03		

### Severity-based subgroup analysis

When participants were stratified by ASD severity using Childhood Autism Rating Scale (CARS) scores, modest differences emerged, though these analyses were limited by small subgroup sizes. In the mild/moderate ASD group (CARS < 36, n = 27 paired comparisons), IgA levels in the ASD children were numerically higher than in their control siblings (2388.19 vs. 1511.38 ng/mg), although this difference was not statistically significant. The S100A8/S100A9 ratio in this subgroup did not differ significantly (0.72 vs. 0.78; p = 0.78; **Table 5**).

**Table 5:** Cal and IgA component ratios in ASD children with CARS scores up to 36 in comparison to their siblings.

Protein ratio	Control group	ASD group	N	p value
S100A8/S100A9			17	0.78
Mean (SD)	0.71 (0.16)	0.74 (0.17)		
Median (Q1, Q3)	0.78 (0.58, 0.81)	0.72 (0.63, 0.84)		
Min - Max	0.38 - 0.93	0.44 - 0.97		
IgA1/IgA2			27	0.49
Mean (SD)	0.44 (0.27)	0.39 (0.29)		
Median (Q1, Q3)	0.45 (0.23, 0.65)	0.41 (0.19, 0.53)		
Min - Max	0.01 - 0.87	0.01 - 0.97		

These analyses were exploratory and *post-hoc*. P-values are unadjusted; false discovery rate (FDR)-adjusted p-values for severe ASD subgroup: S100A8/S100A9 ratio FDR-adjusted p = 0.04. Given small sample sizes (mild/moderate n=17, severe n=11) and multiple comparisons, findings should be considered hypothesis-generating.

**Figure 2:**
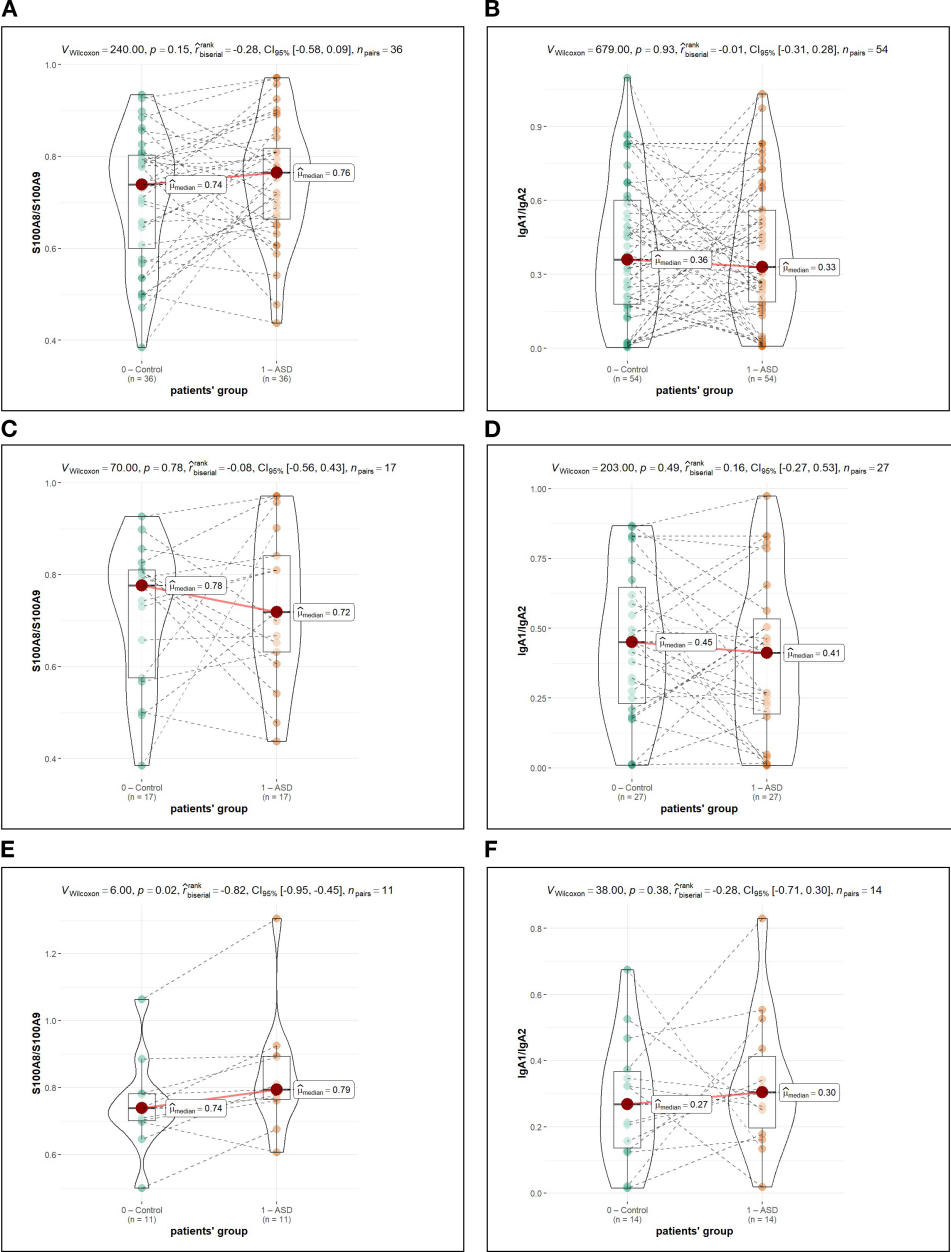
Exploratory analysis of protein ratios stratified by ASD severity (CARS score). Violin plots showing S100A8/S100A9 ratios **(A, C, E)** and IgA1/IgA2 ratios **(B, D, F)** for the full cohort **(A, B)**, mild/moderate ASD subgroup (CARS<36, **(C, D)**), and severe ASD subgroup (CARS>36.5, **(E, F)**. In each panel, 0, control group (siblings); 1, ASD group. The S100A8/S100A9 ratio was significantly elevated in the severe ASD subgroup (panel E: median 0.79 vs. 0.74, unadjusted p=0.02, FDR-adjusted p=0.04, effect size r=-0.82). However, this finding is based on only 11 paired comparisons and should be considered hypothesis-generating pending validation in larger cohorts. All values are dimensionless ratios.

In the exploratory analysis of the severe ASD subgroup (CARS > 36.5, n = 11 paired comparisons), the S100A8/S100A9 ratio showed elevation in children with ASD compared to controls (0.84 vs. 0.75; unadjusted p = 0.02; FDR-adjusted p = 0.04; effect size r = -0.82; **Table 6**, **Figure 2**). However, this finding must be interpreted with substantial caution given the very small sample size, *post-hoc* nature of the analysis, and limited statistical power (estimated power ~18% to detect medium effects). With only 11 paired observations and multiple comparisons across severity strata and protein markers (>28 statistical tests total), the likelihood of false-positive findings is elevated. While this finding remained significant after false discovery rate correction, it should be considered hypothesis-generating and requires validation in adequately powered, pre-registered studies with minimum 50+ pairs per severity category.

**Table 6:** Cal and IgA component ratios in ASD children with CARS scores above 36.5 in comparison to their siblings.

Protein ratio	Control group	ASD group	N	p value
S100A8/S100A9			11	0.02
Mean (SD)	0.75 (0.14)	0.84 (0.18)		
Median (Q1, Q3)	0.74 (0.70, 0.78)	0.79 (0.76, 0.89)		
Min - Max	0.50 - 1.06	0.61 - 1.31		
IgA1/IgA2			14	0.38
Mean (SD)	0.28 (0.19)	0.33 (0.21)		
Median (Q1, Q3)	0.27 (0.14, 0.37)	0.30 (0.20, 0.41)		
Min - Max	0.01 - 0.67	0.02 - 0.83		

These analyses were exploratory and *post-hoc*. P-values are unadjusted; false discovery rate (FDR)-adjusted p-values for severe ASD subgroup: S100A8/S100A9 ratio FDR-adjusted p = 0.04. Given small sample sizes (mild/moderate n=17, severe n=11) and multiple comparisons, findings should be considered hypothesis-generating.

Given the exploratory nature of these severity-stratified analyses, the small subgroup sizes, and multiple testing concerns, all severity-based findings should be interpreted as preliminary observations requiring replication rather than definitive conclusions about ASD severity and inflammatory markers.

## Discussion

### Overall interpretation of primary findings

The present study found no statistically significant differences in faecal levels of α1-antitrypsin, immunoglobulin A (total or subclasses), or calprotectin between children with ASD and their healthy siblings. This null finding is consistent with recent systematic reviews and meta-analyses that have not identified robust evidence of elevated gastrointestinal inflammation in ASD populations. A recent meta-analysis of faecal calprotectin in autism (18) concluded that evidence does not support higher gastrointestinal inflammation in autistic children and adolescents compared to non-autistic controls, though the authors acknowledge substantial heterogeneity among studies and the possibility that a subset of individuals with autism may exhibit elevated gastrointestinal inflammation. Our results contribute to this literature by suggesting that stool inflammatory protein markers may have limited utility as diagnostic biomarkers or stratification tools for ASD in general populations.

### Individual marker interpretation

Immunoglobulin A and Subclasses. While total IgA levels trended higher in the ASD group (p = 0.14), this difference was not statistically significant. This result contrasts with some prior reports. Rose et al. (12) found significantly elevated faecal IGHA1 in 53 children with ASD compared to 34 healthy controls, coupled with lower IGHA2 levels, leading them to propose that altered IgA subclass ratios reflect differences in gut microbiota and immune function. However, these findings have not been universally replicated; Adams et al. (25) observed no differences in faecal IgA between ASD and control children. Additionally, Wang et al. (26) noted that while IgA alterations were detected in some ASD populations, IgA levels appear more indicative of microbiome composition changes than distinguishing autism itself from gastrointestinal symptoms. Our IgA1/IgA2 ratio (0.36 in controls vs. 0.33 in ASD) was virtually identical between groups, and notably, none of the prior studies had specifically examined this ratio. The lack of IgA subclass ratio differences in our sibling-matched design suggests that shared genetic and environmental factors within families may substantially account for IgA dynamics, limiting its utility as an independent biomarker.

Alpha-1-Antitrypsin. We observed slightly lower A1AT-1 levels in the ASD group (142.91 vs. 141.02 ng/mg), though this difference was not significant (p = 0.46). Faecal A1AT-1 is commonly used as a marker of protein-losing enteropathy and gastrointestinal protein leakage. Previous work by Kelly et al. (22) identified associations between A1AT deficiency and autism; notably, Walker-Smith and Andrews (41) observed low serum A1AT in family members of individuals with autism, consistent with a possible familial pattern. More recently, Russo and colleagues (42) reported low serum A1AT levels in family members of people with autism, particularly in those carrying the PiMZ genotype. Our findings do not reveal clinically significant deficiency in either group, though the numerically slightly lower values in ASD warrant further investigation in larger cohorts.

S100A8, S100A9, and Calprotectin. Individual S100A8 and S100A9 levels, as well as total calprotectin, were comparable between groups. Calprotectin, a heterodimeric complex of S100A8 and S100A9, is released upon neutrophil activation and serves as a marker of intestinal inflammation-driven leaky gut. Research on S100A8 and S100A9 in neurologic diseases, including autism, remains limited compared to studies in classical inflammatory conditions. Ashwood et al. (43) reported elevated serum (not faecal) S100A8/A9 levels in children with ASD that correlated with autistic symptom severity, and Peça et al. (44) found increased mRNA expression of S100A8 and S100A9 in peripheral blood mononuclear cells of children with ASD. However, direct comparisons between serum and faecal compartments are limited by different physiological roles and accessibility. Our faecal findings indicate that neutrophil-driven intestinal inflammation as reflected by these markers is not substantially elevated in ASD children compared to their siblings. The ratio of S100A8/S100A9 concentrations may vary across physiological and pathological conditions and can provide insight into inflammatory processes. In the overall comparison, the ratios were nearly identical (0.71 vs. 0.75, p = 0.15), suggesting consistent neutrophil involvement across both groups if present at all.

### Subgroup analysis interpretation

When stratified by ASD severity based on CARS scores, exploratory observations emerged. In the moderate ASD group, IgA levels appeared numerically higher, suggesting possible differences in mucosal immune responsiveness. In the severe ASD group (n = 11), the S100A8/S100A9 ratio was significantly elevated in children with severe autism (p = 0.02). This finding is intriguing but must be interpreted with substantial caution: the severe ASD subgroup comprised only 11 paired individuals, representing only 16% of the total sample. Given the multiple comparisons conducted (seven primary parameters plus ratios across two severity groups), the likelihood of chance findings increases appreciably. Additionally, *post-hoc* subgroup analysis was not pre-registered and represents exploratory investigation. A subset analysis of complete sibling pairs examined with paired statistical tests yielded results largely consistent with the unpaired analysis presented, providing some reassurance regarding robustness despite the limited sample size. Future studies with substantially larger sample sizes in predefined severity categories are needed to determine whether the S100A8/S100A9 finding represents a true biological signal or a statistical artefact. Furthermore, the elevated Type I error risk is compounded by the *post-hoc* nature of the severity stratification and the large total number of statistical tests performed across all comparisons (>28 tests including seven primary markers, two ratios, and three severity strata). Even with FDR correction, the probability that a single significant finding emerges by chance from this many tests remains non-trivial. We urge readers to interpret the S100A8/S100A9 ratio finding with appropriate skepticism until it is replicated in independent cohorts with pre-specified hypotheses and sample sizes of at least 50 pairs per severity category.

### Pathophysiological significance of altered S100A8/S100A9 ratio

S100A8 and S100A9 primarily exist as a stable heterodimer (calprotectin), which constitutes approximately 45% of cytoplasmic proteins in neutrophils and serves as a critical modulator of inflammatory responses (45, 46). The heterodimer acts as a damage-associated molecular pattern (DAMP) that activates toll-like receptor 4 (TLR4) and the receptor for advanced glycation end products (RAGE), triggering downstream NF-κB and MAPK signaling pathways and promoting secretion of pro-inflammatory cytokines including TNF-α and IL-6 (46, 47). Importantly, the stoichiometric balance between S100A8 and S100A9 has distinct functional consequences: in acute myeloid leukemia models, a higher S100A9-to-S100A8 ratio induces cellular differentiation via TLR4-MAPK/ERK-JNK signaling, while excess S100A8 prevents differentiation and maintains an immature phenotype, indicating that the ratio—not merely the total concentration—determines biological outcome (46).

In neurological contexts, S100A8/A9 has been identified as a significant neuroinflammatory marker. The complex activates microglial cells via TLR4 and RAGE, promoting neuroinflammation, and S100A8 aggregation has been shown to precede amyloid-β deposition in Alzheimer’s disease models, establishing a positive feedback loop that exacerbates neurodegeneration (48, 49). Directly relevant to ASD, Boso et al. (50) reported significantly elevated plasma S100A9 levels alongside decreased endogenous secretory RAGE (esRAGE) in young adults with ASD compared to age- and sex-matched controls, with S100A9 levels positively correlated with CARS severity scores (r = 0.49, p = 0.035) (50). More recently, microglia-derived S100A9 has been shown to trigger neuroinflammation cascades, myelination deficits, and social dysfunction in animal models (51), processes directly relevant to ASD pathophysiology (51).

Our finding of an elevated S100A8/S100A9 ratio in the severe ASD subgroup (0.84 vs. 0.75, p = 0.02) may reflect a shift in calprotectin subunit stoichiometry at the intestinal mucosal level. A relative increase in S100A8 compared to S100A9 could alter the balance between the heterodimer’s pro-inflammatory and regulatory functions, potentially modifying downstream TLR4/RAGE signaling at mucosal surfaces. However, it must be emphasized that this interpretation is speculative given the small sample size (n = 11 pairs), the *post-hoc* nature of the analysis, and the inability to determine whether fecal subunit ratios reflect systemic or exclusively local intestinal processes. Validation in adequately powered studies with concurrent measurement of fecal and plasma S100A8/S100A9 is essential before mechanistic conclusions can be drawn.

Beyond their local mucosal effects, S100A8/A9 complexes are increasingly implicated as molecular mediators along the microbiome–gut–mucosal–immune–brain axis in ASD. These proteins exhibit amyloidogenic properties and can interact with other amyloid species, providing a potential mechanistic bridge between intestinal inflammatory signals, systemic neuroinflammation, and CNS circuit alterations that characterize at least a subset of individuals with ASD (52). Although our stool-based measurements cannot directly address CNS processes, they underscore the need for future studies that jointly assess mucosal and central S100A8/A9-related pathways.

## Strengths and limitations

### Strengths

Our study has several notable strengths. First, the sibling-matched design provides superior control for shared genetic background and environmental factors compared to unrelated case-control designs. Second, our rigorous screening protocol systematically excluded known confounders (antibiotics within 6 months, active GI symptoms, dietary abnormalities), enhancing internal validity. Third, we employed state-of-the-art UHPLC-MS/MS methodology with stable isotope-labeled internal standards, ensuring high analytical precision (mean CV 12.9%). Fourth, our study contributes valuable null findings that align with recent meta-analyses, helping to resolve inconsistent literature and prevent publication bias.

### Limitations

Despite these strengths, several important limitations must be acknowledged:

1. Sex Imbalance

A critical limitation is the substantial sex imbalance between groups: 84.2% of the ASD group was male compared to 50.9% of the sibling control group. This imbalance reflects the well-established male predominance in ASD diagnoses (approximately 4:1 ratio) and is inherent to our sibling-matched design. Sex-specific differences in gut microbiota composition, immune function, and metabolic profiles have been documented and are increasingly recognized as important in ASD research. In the maternal immune activation (MIA) mouse model, gut microbiota transplantation from MIA males into healthy males was most effective in reproducing ASD-like symptoms, producing greater intestinal inflammation and gut dysbiosis than female-derived microbiota, suggesting sex-specific gut-immune reactivity contributes to the male bias in ASD (53). Known sex differences in immune function include differential cytokine profiles, innate and adaptive immune cell populations, and IgA dynamics, all of which could influence fecal inflammatory markers independently of ASD diagnosis (47, 54). In our study, the sex imbalance means that observed (or null) differences between ASD and control groups could be confounded by sex-related immune variation rather than reflecting ASD-specific pathology. Although exploratory sex-stratified analyses were conducted, these were substantially underpowered, particularly for females (n = 9 ASD vs. 28 siblings, estimated <10% power). Sex-stratified results are available from the corresponding author upon reasonable request but are not presented due to insufficient statistical power. The sex distribution difference may confound our primary findings and limits generalizability, particularly to females with ASD. Future studies should employ sex-matched sibling pairs or include sex as a covariate in multivariable models (e.g., LMMs) to statistically adjust for this imbalance.

2. Limited Statistical Power for Subgroups

With 57 paired observations, the study had approximately 70% power to detect medium effect sizes but was underpowered (<30% power) to detect small effects. The severity-stratified subgroup analyses were critically underpowered: the mild/moderate subgroup (n=27 pairs) had ~35% power for medium effects, while the severe subgroup (n=11 pairs) had only ~18% power. The sole statistically significant finding (elevated S100A8/S100A9 ratio in severe ASD) emerged from exploratory, *post-hoc* analysis with multiple comparisons, substantially increasing Type I error risk. While the finding remained significant after FDR correction (adjusted p = 0.04), it should be considered hypothesis-generating.

3. High Biological Variability

The high inter-individual variability observed (CVs ranging from 45-101% across markers) likely reflects a combination of true biological variation, dietary factors, circadian timing of collection, and variable home-storage conditions. While analytical precision was excellent (intra-assay CV 12.9%), biological variability substantially exceeded technical variability. This high variability may have reduced our ability to detect true differences between groups.

4. Home-Based Sample Collection Variability

Stool samples were collected by families at home with written instructions, introducing potential variability in collection technique, timing, and storage. While we limited pre-freezing time to maximum 48 hours at -20°C, we cannot quantify the specific contribution of variable compliance to overall measurement variability. Clinic-based supervised collection would minimize this source of variability.

5. Cross-Sectional Design

The cross-sectional design precludes assessment of temporal relationships between inflammatory markers and ASD symptom trajectories. We cannot determine whether inflammatory differences (if present) precede, accompany, or follow ASD symptoms. Longitudinal repeated-measures designs are needed.

6. Age Difference Between Groups

The ASD group was significantly older than siblings (mean difference 1.34 years, p=0.01). While this age difference is modest, it could potentially confound results if inflammatory marker levels change developmentally. We did not conduct age-adjusted analysis because: (1) the age difference was modest (mean 1.34 years, SD 3.49 years), representing typical sibling age spacing, (2) published normative data indicate minimal clinically relevant age-related changes in fecal inflammatory proteins beyond the first year of life. Specifically, for fecal calprotectin, Orfei et al. (55) analyzed 2,788 paediatric samples and found that while infants (<1 year) had significantly elevated levels, the median FCP values for children aged 1–5 years (55 μg/g), 6–14 years (41 μg/g), and 15–16 years (47 μg/g) were not clinically different, with no statistically significant difference between the 6–14 and 15–16 year groups (p = 0.080) (55). Velasco Rodríguez-Belvís et al. (56) reported similar age-related stabilization, with median FC values of 46 μg/g (4–7 years), 34.5 μg/g (8–11 years), and 30 μg/g (12–18 years) (56). Davidson and Lock (57) found no significant difference in median FC between children aged 4–17.9 years (62 μg/g) and adults (61 μg/g) (57). For fecal α1-antitrypsin, Tangsilsat et al. (58) demonstrated stable low levels (median 1.23 mg/dL) in healthy children aged 1 month to 15 years, with no significant age-related variation after the neonatal period (58). While comparable age-specific normative data for fecal IgA subclasses in healthy paediatric populations remain limited, the stability of calprotectin and A1AT across the age range of our study population (predominantly 4–17 years) supports our rationale, and (3) the paired sibling design inherently controls for developmental stage differences within families, as siblings share similar household environments and developmental contexts. Nevertheless, we cannot entirely exclude the possibility that subtle developmental changes in gut immune maturation or age-related differences in gut microbiota composition contributed to observed trends. Future studies could employ age-matched unrelated controls or statistical adjustment using age as a continuous covariate to further isolate ASD-specific effects from developmental changes.

7. Single-Center Recruitment

Our cohort was recruited from a single center in Slovenia, potentially limiting generalizability to other geographic regions, ethnicities, and healthcare systems. Additionally, our stringent exclusion criteria may have selected a relatively “healthy” ASD population.

8. Single-Timepoint Assessment

Inflammatory markers were measured at a single timepoint, precluding assessment of intra-individual variability over time. Repeated measurements would strengthen conclusions.

9. Incomplete Phenotyping

We did not collect detailed dietary records, standardized GI symptom inventories, or information on specific dietary interventions. Such data would enable identification of clinically meaningful subgroups with distinct inflammatory profiles.

10. *Post-Hoc* Exploratory Analyses

We did not collect detailed dietary records, standardized GI symptom inventories, or information on specific dietary interventions beyond our exclusion criteria. Such comprehensive phenotypic data would enable identification of clinically meaningful subgroups with distinct inflammatory profiles. Specifically, we did not employ a validated GI symptom inventory (e.g., Gastrointestinal Severity Index, Rome IV diagnostic questionnaire for functional gastrointestinal disorders), relying instead on systematic parental interview regarding the presence or absence of digestive problems. Recently, validated instruments specifically designed for assessing GI symptoms in ASD populations have become available. Martínez-González et al. (59) developed the Gastrointestinal Symptom Severity Scale (GSSS) based on Rome IV criteria, which showed good internal consistency and test-retest reliability in 265 individuals with ASD, with approximately one-third evidencing clear GI difficulties (59). Holingue et al. (60) developed the ASD Gastrointestinal and Related Behaviors Inventory (ASD-GIRBI), a 36-item psychometrically validated tool that captures both GI symptoms and ASD-specific mealtime behaviors (60). Future studies should employ such validated instruments to enable precise phenotype-inflammatory marker correlations and to identify whether inflammatory markers are elevated specifically in the ASD subgroup with concurrent GI pathology. This approach may have resulted in variable interpretation of “GI symptoms” across families and limited our ability to detect subtle differences in symptom severity. Additionally, while we excluded children with highly restrictive elimination diets (≥2 food groups excluded simultaneously), we did not systematically collect data on macronutrient composition, fiber intake, or specific food sensitivities that could influence gut inflammatory markers. Future studies should employ: (1) validated, standardized GI symptom assessment tools with severity scoring, (2) quantitative 3-day food frequency questionnaires or dietary recalls, (3) comprehensive assessment of supplement use (probiotics, prebiotics, omega-3 fatty acids), and (4) detailed medication history beyond our exclusion criteria to enable more precise phenotype-inflammatory marker correlations and identification of ASD subgroups with distinct gut inflammatory profiles.

### Biological significance and mechanistic implications

Our null findings in a sibling-matched cohort align with recent microbiome research suggesting that the ASD-associated microbial functional architecture identified in case-control studies does not persist in sibling comparisons (16). This pattern suggests that genetic and shared environmental factors substantially shape both microbiota composition and inflammatory responses within families. When comparing unrelated ASD children to neurotypical controls, as in many prior studies, observed differences may partly reflect familial or population-level factors rather than autism-specific pathways. The gut-brain axis involves complex bidirectional signaling through microbial metabolites, bacterial lipopolysaccharides, tight-junction integrity, and immune cell populations. While pro-inflammatory cytokines have been shown to correlate with specific microbial taxa in ASD (16) and may associate with particular autism symptoms (17), the relationship between these systemic immune signals and faecal protein markers of local intestinal inflammation remains incompletely understood. It is possible that alterations in systemic immune responses or specific cytokine patterns may not be reflected in detectable changes in faecal structural proteins like IgA or calprotectin.

Our findings support the emerging consensus that gastrointestinal inflammation, as measured by traditional stool inflammatory markers, is not a universal feature of autism. This does not preclude the possibility that a subset of individuals with autism and comorbid gastrointestinal symptoms may exhibit altered markers; however, population-level screening approaches using these proteins appear of limited value.

### Future directions: multi-omics integration

Future research should adopt multi-omics integration approaches to comprehensively characterize gut-immune-brain axis perturbations in ASD:

1. Microbiome Profiling Integration

Integration of 16S rRNA sequencing or shotgun metagenomics with inflammatory proteins would clarify whether specific bacterial taxa drive local immune responses. Recent work by Morton et al. (16) demonstrated that ASD-microbiome associations observed in unrelated case-control studies do not persist in sibling comparisons, suggesting familial factors substantially shape microbiota. Linking inflammatory proteins to microbiome composition within sibling pairs could disentangle ASD-specific from familial microbial influences.

2. Metabolomics Integration

Measurement of bacterial metabolites (short-chain fatty acids, indole derivatives, p-cresol, trimethylamine N-oxide) alongside inflammatory proteins would reveal functional consequences of microbial activity. Our research group has previously shown altered urinary uremic toxin profiles in ASD children (61, 62), and integrating fecal and urinary metabolomes may identify systemic versus local gut perturbations.

3. Host Transcriptomics and Proteomics

RNA-seq of intestinal epithelial cells from minimally invasive rectal biopsies, combined with expanded proteomic profiling, could reveal mechanistic pathways linking inflammation to barrier dysfunction. Measurement of tight junction proteins (zonulin, occludin, claudins), antimicrobial peptides, and mucin expression would complement our IgA and calprotectin findings.

4. Immune Cell Profiling

Flow cytometric or mass cytometry analysis of stool or peripheral blood immune cell populations, combined with our protein measurements, would clarify whether altered inflammatory proteins reflect cell-type-specific activation patterns.

5. Longitudinal Multi-Omics

Repeated sampling across development (e.g., every 6 months for 2–4 years) with integrated omics would identify critical temporal windows and establish temporal precedence for causal inference.

Study Design Recommendations:

- Large sample sizes: Minimum 200+ sibling pairs
- Sex-matched design
- Pre-registered analysis plans
- Standardized phenotyping with validated GI symptom inventories
- Computational integration methods
- Replication cohorts in diverse populations

Taken together, our findings align with recent reviews of the microbiome–gut–immune–brain axis in ASD, which conclude that while gut dysbiosis, immune activation, and GI comorbidities are clearly present in subgroups, robust stool protein biomarkers capable of distinguishing unselected ASD cohorts from controls have not yet emerged (52, 63, 64). In this context, the largely null group differences observed in our carefully screened sibling-matched sample provide important confirmatory evidence that faecal A1AT, IgA, and calprotectin—at least as measured in broadly ascertained ASD populations without active GI symptoms—are unlikely to serve as stand-alone diagnostic markers.

Future biomarker research in ASD should therefore move beyond single-marker approaches and towards integrated, multi-omic frameworks that combine stool microbiota, metabolomic, and proteomic profiles with validated GI symptom scales and detailed clinical phenotyping (43, 51, 52, 64–66). In particular, parallel assessment of mucosal markers (including S100A8/S100A9 ratios), systemic neuroinflammatory indicators such as circulating S100A9, and longitudinal symptom trajectories may help identify biologically coherent subgroups in whom gut-targeted interventions are most likely to be effective.

## Conclusions

In our sibling-based study of ASD children carefully screened to exclude active gastrointestinal symptoms, we found no statistically significant differences in faecal α1-antitrypsin, IgA, or calprotectin compared to their unaffected siblings. These findings apply specifically to ASD children without clinically apparent GI pathology and cannot exclude the possibility that inflammatory markers differ in the subset of ASD children with concurrent gastrointestinal symptoms or subclinical GI inflammation. When considering mild and severe symptoms, some differences are observed but these findings are based on small sample sizes and should be considered exploratory Overall, our results align with recent meta-analyses indicating no consistent evidence of elevated gastrointestinal inflammation in ASD. Our results are consistent with recent reviews concluding that, while gut dysbiosis and immune activation may occur in subgroups of ASD, robust stool protein biomarkers have not yet emerged for unselected ASD populations (18, 67–69). Stool protein markers therefore appear to have limited diagnostic value in ASD, although they may still reflect subtle alterations in mucosal immunity or gut barrier function. Future studies should include larger, sex-matched cohorts, comprehensive data on diet and gastrointestinal comorbidities. Integration with microbial and metabolomic analyses may also provide more complete insights into gut–immune–brain interactions in ASD.

## Data Availability

The mass spectrometry datasets generated and analyzed during the current study are publicly available in the MassIVE repository at https://doi.org/10.6069/y9hr-js96. Statistical analysis code (R scripts using R version 4.3.1 and RStudio version 2023.12.0) and sex-stratified analyses are available from the corresponding author upon request.
