# Supplementary material for: Faecal inflammatory protein markers in children with autism spectrum disorder are comparable to their healthy siblings
Source: Front Psychiatry. 2026 Apr 15;17:1792801. doi: 10.3389/fpsyt.2026.1792801 (PMC13125985; doi:10.3389/fpsyt.2026.1792801)
Supplement: Supplementary file 1 [file SupplementaryFile1.docx]

**Stool Sample Collection Instructions**

**Clinical Institute of Clinical Chemistry and Biochemistry**
Ljubljana, Slovenia

**Purpose**

You have been provided with a stool collection kit for laboratory analysis. Please follow these instructions carefully to ensure the quality and reliability of your sample.

**Contents of the Collection Kit**

The kit includes:

- Four (4) labeled plastic vials
- A sampling spoon
- A cooling gel pack
- Two plastic transport bags

**Before You Begin**

- Ensure the sample is not contaminated with urine or toilet water.
- Plan to freeze the samples immediately after collection.

**Sample Collection Procedure**

1. **Urination** - The child should urinate into the toilet before stool collection.
2. **Stool Collection** - The child should pass stool onto clean toilet paper.
   *Do not collect stool from the toilet bowl.*
3. **Sample Transfer**
   - Using the provided spoon, transfer a portion of stool into one vial.
   - Fill each vial to approximately one-third of its volume.
   - Repeat the procedure to fill all four (4) vials using stool from approximately the same area.
4. **Sealing and Storage**
   - Close all vials securely.
   - Place the vials into one plastic bag, then place this bag into the second bag.
   - Add the cooling gel pack.
   - **Immediately place the samples in a household freezer.**
5. **Disposal**
   Dispose of the sampling spoon appropriately after use.
6. **Documentation**
   - Record the **date and time of collection** on the accompanying form.
   - Indicate the **stool consistency** by selecting the appropriate option.

**Transport Instructions**

- Transport the samples **frozen and on ice** in a cooling bag.
- Deliver the samples to one of the following:

**Laboratory:**
Clinical Institute of Clinical Chemistry and Biochemistry (KIKKB)
Polyclinic, Njegoševa 4
Corridor A, 1st Floor, Room 11

**or**

**Outpatient Clinic:**
Bring the samples on the day of your scheduled appointment.

**Important:**

- Samples must remain frozen at all times.
- **Do not allow the samples to thaw.**

**Additional Requirements**

Please complete the questionnaire form and bring it with you together with the samples.

**Contact Information**

For any questions or additional information, please contact the laboratory at:
T: +386 1 522 2334
W: [www.kclj.si](http://www.kclj.si)

**Study Context**

This sampling is part of a study on:
**Microbiota, dysbiosis, and intestinal permeability in children with autism spectrum disorder (ASD).**
