# Supplementary material for: Faecal inflammatory protein markers in children with autism spectrum disorder are comparable to their healthy siblings
Source: Front Psychiatry. 2026 Apr 15;17:1792801. doi: 10.3389/fpsyt.2026.1792801 (PMC13125985; doi:10.3389/fpsyt.2026.1792801)
Supplement: Supplementary file 2 [file SupplementaryFile2.docx]

**Supplementary Table 2**

*Chemicals*

Stable isotope labelled [13C6] indole-3-acetic acid (cat. #0317333) was purchased from OlChemIm s.r.o. (Olomouc, Czech Republic). Isotope labelled [13C11] [15N2] L-tryptophan (cat. #574597), purity ≥98%, was purchased from Sigma Aldrich (St. Louis, USA). Isotope labelled [2D4] L-kynurenine (cat. #DLM-7842-PK), purity of 95%, was purchased from Cambridge Isotope Laboratories, Inc. (Tewksbury, Massachusetts, USA). Isotope labelled [13C6] anthranilic acid (cat. #PR-24225), purity 99%, was purchased from Sigma Aldrich (St. Louis, Massachusetts, USA). The chemical standard of L-tryptophan (cat. #51145) (TraceCERT®), N-acetyl-tryptophan (cat. #PHR1177), indole-3-acetate (cat. #45533), purity 98%, L-kynurenine (cat. #K8625), purity ≥98%, were purchased from Sigma Aldrich (St. Louis, Massachusetts, USA). The chemical standard indole-3-carboxaldehyde (cat. #A15330), purity 99%, was purchased from Alfa Aesar (Haverhill, Massachusetts, USA). The standard of indole-3-lactic acid (≥97%) (cat. #SC-255130), purity ≥97%, was purchased from Santa Cruz Biotechnology (Dallas, Texas, USA).

SIL peptide standards with C-terminal arginine (R*; 13C6H14O215N4; + 10 Da mass shift) or lysine (K*; 13C6H14O2 15N2; + 8 Da mass shift) were custom synthesized (JPT Technologies, Berlin,Germany). SIL synthetic peptides extended with 3 natural aminoacid residues at C-terminus and N-terminus of peptide. Trypsin gold was from Promega (cat. #V5280).

Liquid chromatography-mass spectrometry (LC-MS) grade acetonitrile (ACN) (cat. #0012078) was purched from Biosolv. LiChrosolv isopropanol (IPA) (cat. #102781) was purchased from Supelco. Formic acid (cat. #A117-50) and 2-iodoacetamide (cat. #A14715) were purchased from Fisher Scientific (Pardubice, Czechia). Ammonium bicarbonate (cat. #09830) was from Sigma Aldrich (St. Louis, MO). Sodium deoxycholate (cat. #3484.1) and 1,4-dithiothreitol (cat. #6908.1) were purchased from Carl Roth (Karlsruhe, Germany). BCA protein assay kit (cat. #23225) was purchased from Thermo Fisher Scientific (Waltham, MA). The deionized water was produced in Sartorius Aurum Mini ultrapure water system (Göttingen, Germany).

*Stool sample collection*

Stool samples were removed from -80 °C freezer and thawed on ice for 1 hour. Thawed stool samples were collected using a pre-weighed broken FLOQswab (Copan Diagnostics, USA, cat# 520CS01) into a homogenization vial (Benchmark Scientific, USA, cat# D1031-T20) and weight of sample measured. Samples were closed with aluminum foil and placed into -80 °C for 30 min prior to freeze drying overnight. Dried stool samples were weighed, sealed with screw lid, and kept at -80 °C until extraction. The wet weight, dry weight, and water content of stool samples were calculated.

*Protein analysis - stool sample preparation*

Proteins were extracted from dried stool sample using protein extraction buffer content of 50 mM Ammonium bicarbonate with 5g/L sodium deoxycholate. 1 mL of protein extraction buffer was added to vial and proteins were extracted using a homogenizer with setting: 4 pulses × 10 s; 4 m/s; inter-time 10 s; lab temperature. Extracts were centrifuged at 13 000 RPM for 5 min at 8 °C and 500 µL supernatant transferred to a new 96-deep well plate (Costar, cat. #3959) and stored at -80 °C until further analysis.

The 96-well plate with protein extract was taken out of the freezer and thawed at RT for 90 min. Total protein concentration was measured using BCA kit (Thermo Scientifics, cat# 23225). Samples were diluted 10-fold before BCA analysis via taking 10 μL into a microplate and adding 90 μL of protein extraction buffer. Samples were diluted to achieve total protein concentration up to 250 μg/sample. Thus a 50, 30 or 20 μL of protein extract was taken for following sample preparation. Extraction buffer was added to samples with 20 and 30 μL to achieve a volume 50 μL for all samples. Proteins in the samples were then reduced using 5 μL of 200 mM dithiothreitol for 10 min at 90 °C. After cooling, the proteins were alkylated using 5 μL of 400 mM iodoacetamide and incubated for 30 min in dark. A 10 μL of 500 nM SIL peptides mixture was then added to sample. A list of SIL peptides is shown in **Table B**. Reduced and alkylated proteins were digested adding 3 μL of 1 μg/μL trypsin solution. Samples were properly sealed and placed into shaking incubator heated at 37 °C for 5 hours (Biosan, Latvia, ES-20). The digestion was stopped adding 200 μL of 2% formic acid. Digested samples were processed at SPE Oasis prime HLB 96 well plate, 30 mg (Waters, USA, cat# 186008054). Samples were directly loaded on SPE and washed with 300 μL of 2% FA. Peptides were eluted into a new 96 well plate with V bottom (Waters, cat.#186005837) using 50% ACN with 2% FA. Then the samples were dried out in vacuum evaporator and dissolved in 5% ACN with 0.1% FA.

| Protein Number | Gene Name | Name | Abbevariation | Peptide Sequence |
| --- | --- | --- | --- | --- |
| P01009-1 | SERPINA1 | Alpha-1-antitrypsin isoform 1 | A1AT-1 | AVLTIDEK |
| P01876 | IGHA1 | Immunoglobulin heavy constant alpha 1 | IGHA1 | TPLTATLSK |
| P01877/P01876 | IGHA1+2 | Immunoglobulin heavy constant alpha 1 and 2 | IGHA1+2 | WLQGSQELPR |
|  |  |  |  | SAVQGPPER |
| P01877 | IGHA2 | Immunoglobulin heavy constant alpha 2 | IGHA2 | DASGATFTWTPSSGK |
| P12724 | RNASE3 | Eosinophil cationic protein | ECP | NQNTFLR |
| P10153 | RNASE2 | Eosinophil-derived neurotoxin | EDN | DPPQYPVVPVHLDR |
| P05164 | MPO | Myeloperoxidase | MPO | QNQIAVDEIR |
|  |  |  |  | VVLEGGIDPILR |
| P05109 | S100-A8 | Calprotectin 1 | S100-A8 | ALNSIIDVYHK |
|  |  |  |  | GADVWFK |
| P06702 | S100-A9 | Calprotectin 2 | S100-A9 | DLQNFLK |
|  |  |  |  | LGHPDTLNQGEFK |

**Table B** List of SIL peptides in standard mixture

*Mass spectrometry protein analysis*

Samples were analyzed on a UHPLC system (1260 series Agilent, CA) coupled with a triple quadrupole mass spectrometer (AJS 6495A, Agilent, CA). Samples were injected (2 µL) on the analytical column (C18 Peptide CSH; 1.7 µm, 2.1 mm i.d. × 100 mm; cat. #186006937; Waters, MA). The column temperature was held at 40 °C. The mobile phase consisted of solution A (0.1% FA in water) and solution B (0.1% FA in 95% ACN). The flow rate was 300 µL/min. The gradient elution program consisted of analytical (0–30.9 min) and re-equilibration part (31–35 min): 0.0 min 5% B; 25 min 30% B; 25.5 min 95% B; 30.9 min 95% B; 31 min 5% B; 35 min 5% B. A standard-flow electrospray source operated in positive ion mode (capillary voltage 3.5 kV; gas flow rate 18 L/min at 200 °C; sheath gas flow 12 L/min at 350 °C; nozzle voltage 500 V). We monitored 78 transitions per the dynamic SRM mode analysis, with 3 min window scheduled around peptide experimental RT. SRM signature transitions were equivalent for proteotypic peptide and corresponding SIL internal standard, i.e., a single SRM quantifier transition and 2 additional qualifier SRM transitions were acquired (**Table C**).

**Table C:** Transition list of analyzed peptides. Product ion used for peptide quantification is highlighted in bold font.

| Protein | Peptide | Precursor Ion | Product Ion | Qualified/**Quantified** ion |
| --- | --- | --- | --- | --- |
| P05109 | ALNSIIDVYHK.heavy | 640.86 | 669.34 | y5 |
| P05109 | ALNSIIDVYHK.heavy | 640.86 | 782.43 | **y6** |
| P05109 | ALNSIIDVYHK.heavy | 640.86 | 292.19 | y2 |
| P05109 | ALNSIIDVYHK.light | 636.85 | 774.41 | **y6** |
| P05109 | ALNSIIDVYHK.light | 636.85 | 661.33 | y5 |
| P05109 | ALNSIIDVYHK.light | 636.85 | 284.17 | y2 |
| P01009 | AVLTIDEK.heavy | 448.76 | 726.41 | **y6** |
| P01009 | AVLTIDEK.heavy | 448.76 | 613.33 | y5 |
| P01009 | AVLTIDEK.heavy | 448.76 | 399.20 | y3 |
| P01009 | AVLTIDEK.light | 444.76 | 718.40 | **y6** |
| P01009 | AVLTIDEK.light | 444.76 | 605.31 | y5 |
| P01009 | AVLTIDEK.light | 444.76 | 391.18 | y3 |
| P01877 | DASGATFTWTPSSGK.heavy | 760.86 | 871.44 | y8 |
| P01877 | DASGATFTWTPSSGK.heavy | 760.86 | 770.39 | y7 |
| P01877 | DASGATFTWTPSSGK.heavy | 760.86 | 483.27 | **y5** |
| P01877 | DASGATFTWTPSSGK.light | 756.85 | 863.43 | y8 |
| P01877 | DASGATFTWTPSSGK.light | 756.85 | 762.38 | y7 |
| P01877 | DASGATFTWTPSSGK.light | 756.85 | 475.25 | **y5** |
| P06702 | DLQNFLK.heavy | 443.25 | 657.38 | **y5** |
| P06702 | DLQNFLK.heavy | 443.25 | 529.32 | y4 |
| P06702 | DLQNFLK.heavy | 443.25 | 268.21 | y2 |
| P06702 | DLQNFLK.light | 439.24 | 649.37 | **y5** |
| P06702 | DLQNFLK.light | 439.24 | 521.31 | y4 |
| P06702 | DLQNFLK.light | 439.24 | 260.20 | y2 |
| P10153 | DPPQYPVVPVHLDR.heavy | 547.96 | 715.39 | **y12** |
| P10153 | DPPQYPVVPVHLDR.heavy | 547.96 | 477.27 | y12 |
| P10153 | DPPQYPVVPVHLDR.heavy | 547.96 | 373.71 | y6 |
| P10153 | DPPQYPVVPVHLDR.light | 544.62 | 710.39 | **y12** |
| P10153 | DPPQYPVVPVHLDR.light | 544.62 | 473.93 | y12 |
| P10153 | DPPQYPVVPVHLDR.light | 544.62 | 368.71 | y6 |
| P05109 | GADVWFK.heavy | 415.72 | 702.37 | **y5** |
| P05109 | GADVWFK.heavy | 415.72 | 587.34 | y4 |
| P05109 | GADVWFK.heavy | 415.72 | 488.27 | y3 |
| P05109 | GADVWFK.light | 411.71 | 694.36 | **y5** |
| P05109 | GADVWFK.light | 411.71 | 579.33 | y4 |
| P05109 | GADVWFK.light | 411.71 | 480.26 | y3 |
| P06702 | LGHPDTLNQGEFK.heavy | 488.58 | 730.36 | **y6** |
| P06702 | LGHPDTLNQGEFK.heavy | 488.58 | 621.30 | y6 |
| P06702 | LGHPDTLNQGEFK.heavy | 488.58 | 488.26 | y4 |
| P06702 | LGHPDTLNQGEFK.light | 485.91 | 722.35 | **y6** |
| P06702 | LGHPDTLNQGEFK.light | 485.91 | 621.30 | y6 |
| P06702 | LGHPDTLNQGEFK.light | 485.91 | 480.25 | y4 |
| P12724 | NQNTFLR.heavy | 451.74 | 660.37 | **y5** |
| P12724 | NQNTFLR.heavy | 451.74 | 546.33 | y4 |
| P12724 | NQNTFLR.heavy | 451.74 | 298.21 | y2 |
| P12724 | NQNTFLR.light | 446.74 | 650.36 | **y5** |
| P12724 | NQNTFLR.light | 446.74 | 536.32 | y4 |
| P12724 | NQNTFLR.light | 446.74 | 288.20 | y2 |
| P05164 | QNQIAVDEIR.heavy | 598.32 | 712.39 | **y6** |
| P05164 | QNQIAVDEIR.heavy | 598.32 | 641.35 | y5 |
| P05164 | QNQIAVDEIR.heavy | 598.32 | 542.28 | y4 |
| P05164 | QNQIAVDEIR.light | 593.31 | 702.38 | **y6** |
| P05164 | QNQIAVDEIR.light | 593.31 | 631.34 | y5 |
| P05164 | QNQIAVDEIR.light | 593.31 | 532.27 | y4 |
| P01876/P01877 | SAVQGPPER.heavy | 475.75 | 693.36 | y6 |
| P01876/P01877 | SAVQGPPER.heavy | 475.75 | 565.30 | **y5** |
| P01876/P01877 | SAVQGPPER.heavy | 475.75 | 508.28 | y4 |
| P01876/P01877 | SAVQGPPER.light | 470.75 | 683.35 | y6 |
| P01876/P01877 | SAVQGPPER.light | 470.75 | 555.29 | **y5** |
| P01876/P01877 | SAVQGPPER.light | 470.75 | 498.27 | y4 |
| P01876 | TPLTATLSK.heavy | 470.28 | 741.46 | **y7** |
| P01876 | TPLTATLSK.heavy | 470.28 | 628.38 | y6 |
| P01876 | TPLTATLSK.heavy | 470.28 | 527.33 | y5 |
| P01876 | TPLTATLSK.light | 466.28 | 733.45 | **y7** |
| P01876 | TPLTATLSK.light | 466.28 | 620.36 | y6 |
| P01876 | TPLTATLSK.light | 466.28 | 519.31 | y5 |
| P05164 | VVLEGGIDPILR.heavy | 645.89 | 850.50 | **y8** |
| P05164 | VVLEGGIDPILR.heavy | 645.89 | 508.35 | y4 |
| P05164 | VVLEGGIDPILR.heavy | 645.89 | 312.23 | b3 |
| P05164 | VVLEGGIDPILR.light | 640.88 | 840.49 | **y8** |
| P05164 | VVLEGGIDPILR.light | 640.88 | 498.34 | y4 |
| P05164 | VVLEGGIDPILR.light | 640.88 | 312.23 | b3 |
| P01876/P01877 | WLQGSQELPR.heavy | 612.32 | 796.42 | **y7** |
| P01876/P01877 | WLQGSQELPR.heavy | 612.32 | 739.40 | y6 |
| P01876/P01877 | WLQGSQELPR.heavy | 612.32 | 652.37 | y5 |
| P01876/P01877 | WLQGSQELPR.light | 607.32 | 786.41 | **y7** |
| P01876/P01877 | WLQGSQELPR.light | 607.32 | 729.39 | y6 |
| P01876/P01877 | WLQGSQELPR.light | 607.32 | 642.36 | y5 |

*Quality control*

The UHPLC-MS sequence included analysis of instrumental blanks (after every 3 samples) and in lab-made peptide and metabolite mixture QC (approximately after every 30 samples). The average CV of peptides and metabolites peak areas was below 12.9% and 10.5 %, respectively.
